# Effectiveness of integrating patient-reported outcome measures into electronic health records for measurement-based care in patients with chronic illnesses: a systematic review

**DOI:** 10.3389/frhs.2026.1930126

**Published:** 2026-10-14

**Authors:** Monica Cepoiu-Martin, Sabrina Adam, Fakhriyya Aghabayli, Sadia Ahmed, Diane L. Lorenzetti, Lauren Verwaayen, Haleema Yousuf, Maria J. Santana

**Affiliations:** 1 Department of Community Health Sciences, Cumming School of Medicine, University of Calgary, Calgary, AB, Canada; 2 Alberta Strategy for Patient-Oriented Research Support Unit, Calgary, AB, Canada; 3 Health Sciences Library, University of Calgary, Calgary, AB, Canada; 4 Cumming School of Medicine, University of Calgary, Calgary, AB, Canada; 5 Department of Pediatrics, Cumming School of Medicine, University of Calgary, Calgary, AB, Canada

**Keywords:** chronic disease, clinical decision-making, electronic health records, healthcare utilization, patient-reported outcome measures, systematic review, measurement-based care

## Abstract

**Background:**

Measurement-based care (MBC) has emerged as a promising strategy to support effective chronic disease management. Patient-reported outcome measures (PROMs) are increasingly used to capture patients’ perspectives on symptoms and quality of life, and their integration into electronic health record (EHR) systems may enhance their clinical utility. However, evidence on their impact when embedded within routine clinical workflows remains limited.

**Objective:**

To systematically identify and synthesize evidence on the impact of integrating patient-reported outcome measures (PROMs) into electronic health record (EHR) systems on clinical care processes and patient outcomes among individuals with chronic conditions.

**Methods:**

A systematic review was conducted in accordance with PRISMA guidelines. Multiple databases (MEDLINE, Embase, CINAHL, PsycINFO, Cochrane Library, Scopus, and Web of Science) were searched from January 2000 to February 2026. Eligible studies included quantitative research evaluating EHR-integrated PROMs used in routine clinical care for chronic conditions. Data extracted included study characteristics, PROM type, integration approach, and clinical and process-related outcomes.

**Results:**

Of 10 included studies, most were conducted in the United States and spanned oncology, rheumatology, primary care, and musculoskeletal settings. Some of these studies reported that EHR-integrated PROMs improved symptom identification and monitoring and were associated with gains in clinic efficiency, including shorter wait times and fewer follow-up visits. Some studies reported reduced healthcare utilization and increased diagnostic activity. However, effects on patient satisfaction, visit volume, and broader clinical outcomes were inconsistent. Heterogeneity in PROMs, implementation approaches, and outcomes precluded meta-analysis.

**Conclusions:**

EHR-integrated PROMs may improve symptom monitoring and help clinicians identify patient needs more efficiently within routine chronic disease care. Several studies also reported benefits related to clinic workflow, including shorter wait times, fewer follow-up visits, and improved care coordination. However, the impact on patient satisfaction, healthcare utilization, and longer-term clinical outcomes was less consistent across studies. Overall, PROMs appeared to be most useful when they were directly integrated into clinical workflows and used to inform real-time care decisions. Further research in more diverse patient populations and healthcare settings is needed to better understand their long-term effectiveness and real-world use in chronic disease management.

**Systematic Review Registration:**

https://www.crd.york.ac.uk/PROSPERO/view/CRD420261297619, PROSPERO CRD420261297619.

## 1 Introduction

Chronic health conditions represent a major and increasing global health burden, accounting for approximately 41 million deaths annually (1). These conditions are typically long-term and multifaceted, often requiring ongoing monitoring, coordination across multiple healthcare providers, and sustained patient engagement. As a result, managing chronic diseases can be complex for both patients and healthcare systems, particularly in navigating care pathways and addressing diverse and evolving patient needs. Measurement-based care (MBC) has emerged as a promising strategy to support effective chronic disease management by enabling systematic tracking of patient outcomes and data-driven treatment adjustments (2).

Measurement-based care (MBC) is a systematic, patient-centered approach that involves the routine collection of standardized patient-reported outcome measures (PROMs) to monitor symptoms, functioning, and treatment response, with results used to inform shared decision-making and timely treatment adjustments (3, 4). In the context of chronic conditions—such as diabetes, cardiovascular disease, arthritis, chronic pain, and mental health comorbidities—MBC offers particular value by enabling longitudinal tracking of fluctuating symptoms, functional status, and quality of life over extended periods. Over the past decade, evidence has accumulated showing that MBC can improve clinical outcomes in chronic illness populations by facilitating earlier detection of deterioration, enhancing patient engagement and adherence, reducing treatment inertia, and supporting more personalized care plans (2, 5). For example, in diabetes and heart failure management, regular PROMs feedback has been associated with better symptom control, fewer hospitalizations, and improved patient–provider communication (6). Despite these benefits, adoption in routine chronic disease management remains inconsistent due to barriers such as workflow integration, clinician training, and technology limitations, underscoring the need for strategies like EHR-embedded PROMs to scale MBC effectively across diverse chronic care settings.

Patient-reported outcome measures (PROMs) are standardized questionnaires that capture patients' perspectives on their health status, symptoms, functioning, and quality of life (7). PROMs provide structured insight into symptom burden—including severity, frequency, and progression—as well as the impact on daily functioning and overall well-being. The use of PROMs in healthcare has expanded across various settings, with growing interest in incorporating them into routine clinical practice to support patient-centered care and shared decision-making (8–10). At the same time, advancements in digital health technologies have enabled the electronic collection of PROMs, facilitating efficient data capture and longitudinal monitoring (11). Reviews of electronically collected PROMs highlight their potential to enhance care delivery, although implementation varies widely across contexts (12–14).

However, much of the existing literature has focused on the feasibility of PROMs collection rather than their integration into clinical workflows (15). Integrating electronic PROMs (ePROMs) into clinical workflows involves routinely collecting and reviewing patient-reported data as part of standard care, typically by embedding them within electronic health record (EHR) systems. This enables clinicians to access real-time patient-reported information during encounters and incorporate it directly into decision-making. Emerging literature suggests that integrating PROMs directly into EHR systems may enhance their clinical utility by making patient-reported data readily available during encounters and supporting data-driven care processes (16–18). Despite this potential, the evidence on the impact of integrating PROMs into EHR systems on clinical care processes and patient outcomes in chronic illness remains (10) limited, heterogeneous, and scattered across disparate studies (19).

The objective of this systematic review was to identify and synthesize the available evidence on the impact of integrating patient-reported outcome measures (PROMs) into electronic health record (EHR) systems on clinical care systems and processes, as well as patient outcomes, among individuals with chronic conditions.

## 2 Methods

This study followed the PRISMA reporting guidelines (20) and the protocol has been registered with Prospero (CRD420261297619). We conducted a comprehensive literature search to identify studies eligible for inclusion in this systematic review. The search strategy was developed in collaboration with a research librarian (DL) and designed in accordance with established methodological guidance, including the Cochrane Handbook and PRISMA-P recommendations. Electronic databases searched included MEDLINE (Ovid), Embase (Ovid), CINAHL (EBSCO), APA PsycInfo (Ovid), the Cochrane Library (Wiley), Scopus (Elsevier), and Web of Science (Clarivate). The search combined controlled vocabulary terms (e.g., MeSH) and free-text keywords related to patient-reported outcome measures (PROMs), measurement-based care, and electronic health record (EHR) systems. Searches were limited to studies published from January 2000 to February 2026 to reflect the emergence of PROM integration within EHR systems. This window was intentionally broad; eligible studies of PROMs that were both EHR-integrated and used in routine chronic care were expected to cluster in later years as patient portals, interoperable data exchange, and clinical decision-support functions matured. No language restrictions were applied. In addition to database searches, the reference lists of all included studies were manually screened to identify any additional relevant articles. The full search strategies are provided in Supplementary Appendix A.

For this review, studies were considered eligible for inclusion if they met predefined data specifications. Eligible studies included patients receiving care for chronic health conditions. For this review, a PROM was defined as a standardized measure of symptoms, functioning, or health-related quality of life completed directly by the patient, without interpretation by a clinician or proxy (ISOQOL) (21). Instruments were classified as patient-completed PROMs, proxy- or informant-completed measures, composite indices that combine PROM items with clinical data (for example, ASDAS, which includes CRP or ESR), or non-PROM digital tools (for example, EHR-based algorithms). Studies were eligible if the intervention included EHR-integrated patient-completed PROMs in routine care; composite or accompanying non-PROM components were extracted and labelled separatelyThe intervention of interest was the systematic collection and clinical integration of PROMs within EHR systems as part of routine clinical care, consistent with measurement-based care approaches. Eligible designs were original quantitative evaluations of PROMs integrated into an EHR and used in routine clinical care, including randomized and cluster-randomized trials, cohort studies, interrupted time series, pre–post implementation studies, quality-improvement evaluations, and single-arm studies that reported clinical or process outcomes. We excluded studies that assessed only the feasibility of PROM collection or of the instrument itself (acceptability, completion, or validation without EHR-integrated clinical use). Studies labelled “feasibility” were eligible if they evaluated EHR-integrated PROM workflows in routine care and reported downstream clinical or process outcomes (for example, alerts, triage, or clinician action), were included. Studies were required to report at least one relevant outcome, including patient-level outcomes such as health-related quality of life, symptom burden, functional status, clinical indicators, or healthcare utilization, and/or clinical process outcomes such as patient–provider communication, engagement in care, clinical management or treatment decisions, shared decision-making, visit frequency, or hospital readmissions. Additionally, PROMs had to be integrated within EHR systems and used within routine clinical workflows rather than as standalone tools or external tools.

Studies were excluded if they did not meet the above criteria, if the individuals in the study did not have chronic conditions. Study designs such as case reports, case series, cross-sectional studies, surveys, pilot studies, reviews, commentaries, opinion articles, and conference abstracts were excluded. Studies focusing exclusively on acute conditions or conducted outside of routine clinical care settings were also excluded. Articles were excluded if PROMs were not used, were not validated, or were implemented outside of EHR systems, including collection via paper, telephone, email, interviews, REDCap, or other standalone survey platforms. Studies that focused solely on the technical aspects of PROMs implementation, the development or validation of PROMs, or the collection and integration of patient-reported experience measures (PREMs) were also excluded.

All records identified through the database searches and manual reference screening were imported into Covidence, where duplicate records were removed. The study selection process was conducted in two stages. In the first stage, titles and abstracts were independently screened by pairs of reviewers against the predefined inclusion and exclusion criteria in Covidence. Studies deemed potentially relevant by at least one reviewer were then reviewed by the lead investigator, to decide if they advanced to full-text screening. In the second stage, two reviewers independently assessed full-text articles for eligibility through a Covidence template. Discrepancies at both stages were resolved through discussion with a third reviewer, and where consensus could not be reached, a third reviewer adjudicated the final decision. Reasons for exclusion at the full-text stage were documented. Further, three reviewers independently assess the risk of bias of included studies using the mixed methods appraisal tool (MMAT) (22). Discrepancies were resolved in discussions and consulted with a fourth reviewer. The study selection process was reported using a PRISMA flow diagram (Figure 1).

**Figure 1:**
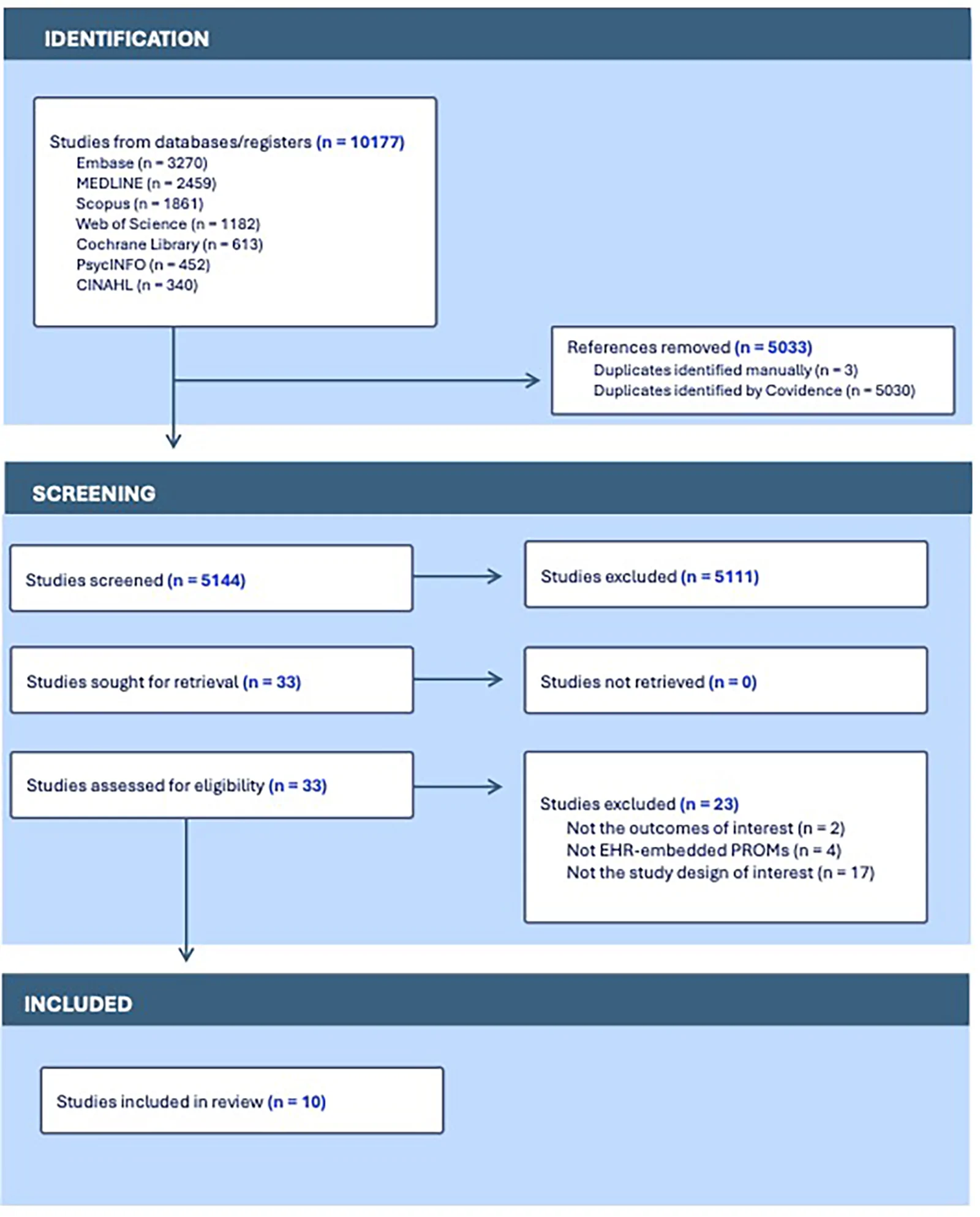
PRISMA systematic review workflow.

Data were extracted using a standardized form and included study characteristics (year of publication, country, and study design), population characteristics (clinical setting and patient population), and details of the PROMs, including measure type and administration. Information on the integration of PROMs into electronic systems or clinical workflows was also collected. Outcomes of interest included patient clinical outcomes, and system and process-related measures, such as healthcare utilization, symptom identification, and workflow efficiency.

When assessing the effectiveness of the integration of PROMs into EHRs, outcomes are often categorized in the literature into three levels: patient, clinician, and system (2–5, 18, 23–25). We summarized the outcomes examined in the studies included in our systematic review according to these levels.

*Patient-Level Outcomes* focus on direct effects on individuals receiving care and may include symptom reduction (e.g., pain, fatigue, depression scores), disease control (e.g., HbA1c, blood pressure), quality of life, and functional status outcomes, satisfaction with care, shared decision-making experience, treatment adherence, and self-management confidence, as well as reduced emergency department visits, hospitalizations, or unplanned care. Others include mortality, health-related quality of life (HRQoL), and patient activation/engagement measures.

*Clinician-Level Outcomes* evaluate changes in provider behavior, experience, and decision-making and may include frequency of PROM review during visits, adherence to MBC protocols or decision-support algorithms, changes in treatment adjustments (e.g., medication titration, referral rates), earlier detection of deterioration, satisfaction with the intervention, perceived usefulness of integrated PROM data, reduced cognitive burden or burnout, and confidence in managing complex chronic cases, improvements in understanding patient perspectives or comfort with data-driven care.

*System-Level Outcomes* assess broader organizational or healthcare system impacts and may include reduced overall healthcare costs, shorter wait times, improved care coordination across providers/settings, reach (proportion of eligible patients receiving the intervention), adoption by clinics, sustainability over time, and fidelity at scale, reduced care fragmentation, fewer adverse events, better equity in outcomes across demographic groups, and compliance with standards (e.g., accreditation metrics), improvements in aggregate chronic disease metrics (e.g., hospital readmission rates for heart failure patients), cost-effectiveness, and health system performance indicators.

Due to heterogeneity in study designs, PROMs used, and outcome measures, a meta-analysis was not conducted. Results were summarized descriptively across studies to identify patterns and trends in the impact of EHR-integrated PROMs on the patient, clinician and system-level outcomes. Moreover, given the scarcity of studies of PROMs that are both EHR-integrated and used in routine chronic care, we included multiple clinical contexts. To avoid treating these as interchangeable, we extracted the purpose of PROM use and the type of EHR integration (passive display vs. alerts/triage/decision support) and synthesized findings by outcome domain and by clinical setting rather than pooling them.

## 3 Results

### 3.1 Study characteristics

A total of 10,177 records were identified across seven databases, with 5,033 duplicates removed, leaving 5,144 records for title and abstract screening. Following title and abstract screening, 5,111 records were excluded, leaving 33 reports for full-text assessment. Of these, 23 reports were excluded based on the predefined eligibility criteria, resulting in 10 studies being included in the systematic review. Most studies (*n* = 9) were conducted in the United States (11, 17, 26–32) with one study conducted in the United Kingdom (33).

Methodological quality was mixed (Supplementary Appendix B). Cluster randomized trials generally had appropriate randomization and comparable groups, but outcome assessors were not blinded. Non-randomized and implementation studies more often had incomplete outcome data, high non-response, limited representativeness, uncontrolled confounding, or uncertain intervention fidelity. These limitations were considered when interpreting effects, particularly for patient-level and longer-term outcomes.

The study characteristics of the studies included in our systematic review are summarized in Table 1. Study designs varied across the included studies. Four studies used cluster randomized controlled trial designs in oncology and primary care settings (26–28, 32). The remaining studies employed a range of methodologies including a controlled interrupted time series (17), an observational longitudinal study (33), and a retrospective cohort study (29), Additional designs included a prospective single-arm feasibility study (30), a pre–post implementation study (11), and a clinical quality improvement initiative (31). All studies were conducted in clinical care settings, predominantly in ambulatory and outpatient environments. Oncology-focused studies were carried out in ambulatory oncology and outpatient cancer settings (26, 27, 29–31). Primary care settings included urban primary care clinics (28) and academic-affiliated family medicine clinics (32). Rheumatology care was delivered in hospital-based outpatient clinics (17, 33), and one study was conducted in a tertiary spine surgery clinic (11) Two studies took place within large academic health systems (17, 26), while one was conducted in community-based settings, including a community cancer center (30). Multi-site cluster trials included 7 clusters across 30 oncology clinics, with 26 included in the final analysis (26). 15 oncology and hematology clinic clusters (26), (9 primary care clinics (28) and 4 family medicine clinics (32). The remaining studies were conducted within single institutions or specialty centers, including a large academic hospital (17), a single hospital in the United Kingdom (33), a comprehensive cancer center (29), a community cancer center (30) oncology outpatient clinics within a single institution (31), and a tertiary spine surgery clinic (11). The included studies focused on adult populations across multiple clinical domains. These included oncology (25, 26, 28–30), primary care (27, 31), rheumatology (17, 32) and spine/surgical specialty care (11).

**Table 1:** Summary of study characteristics and outcomes.

Studies	Country	Study design	Setting	Population	EHR platform	PROMs used	Patient-level outcomes	Clinician-level outcomes	System-level outcomes
Azad et al. (11)	USA	Pre-Post Implementation Study	Tertiary Academic Spine Surgery Clinic, Stanford University, USA	Outpatients	Epic Systems	PROMs used to streamline workflow and assessment	NR	NR	Clinic visit time ↓ by ∼10 min (127.9 to ∼117 min; 10.1% reduction, *p* < 0.0001); PROM capture rate ↑ (∼98%).
Boustani et al. (28)	USA	Cluster randomized controlled trial	9 urban primary care clinics, Indianapolis, Indiana, USA	Outpatients	Epic Systems	PROMs used for screening and guiding diagnostic evaluation	NR	Dementia diagnosis ↑12.4% to 15.4% (AOR=1.31, 95% CI: 1.05 to 1.64); diagnostic evaluations ↑29.0% to 36.7%.	NR
Chan et al. (33)	UK	Observational longitudinal study	Rheumatology clinics, Royal Berkshire Hospital, UK	Outpatients	Oracle Health Millennium (EPR)	ePROMs integrated into EHR with alerts for monitoring and triage	ePROM completion ↑ 25% to 66%; Patient experience: 77%: easy to use 69%: prefer digital	PROM use: 97% reported time savings; 97%–99% reported improved monitoring and usability	↓ wait times (∼50%; 12.1 to 6.4 weeks); ∼1,500 clinic hours saved/year; ↑ capacity (new visits 427 to 3,129); improved N:FU ratio (1:3.1 to 1:2.2)
Cheville et al. (27)	USA	Cluster randomized controlled trial	15 clusters of medical oncology and haematology clinics, USA	Oncology/Hematology	Epic Systems	EHR-integrated PROMs used for symptom monitoring and collaborative care	85% of patients reported at least one actionable symptom; anxiety ↓(−0.12, 95% CI −0.19 to −0.05); depression ↓ (−0.08, 95% CI −0.15 to −0.01). −0.15 to −0.01)	EHR-integrated clinical decision support	↓Hospital admissions: RR = 0.39 (95% CI: 0.38 to 0.42); ↓ ER visits: RR = 0.38 (95% CI: 0.36 to 0.40); ↓ ICU admissions: RR = 0.38 (95% CI: 0.38 to 0.46)
Garcia et al. (31)	USA	Quality improvement implementation study	Medical oncology outpatient clinics at the Robert H. Lurie Comprehensive Cancer Center. Chicago, USA	Oncology outpatient	Epic Systems	PROM-triggered alerts used for identifying care needs	Higher symptom burden among patients with identified needs (no evidence of symptom improvement)	>50% of assessments triggered ≥1 alert; 41.8% identified nutritional needs	Mean total encounters: 13.11 encounters per patient (SD 11.10)
Harle et al. (32)	USA	Cluster randomized controlled trial	4 family medicine clinics affiliated with an academic health center, USA	Primary Care (chronic pain)	Collaborative Health Outcome Information Registry (CHOIR) Systems	PROMs integrated into EHR for routine care	Patient satisfaction: 9.28 vs. 9.01 (*P* = 0.20)	NR	NR
Natori et al. (29)	USA	Retrospective cohort study	Ambulatory oncology clinics at the Sylvester Comprehensive Cancer Center, University of Miami Miller School of Medicine.	Oncology	My Wellness Check (MWC)	PROM completion used for monitoring and care coordination	↓ ER visits (HR 0.78); ↓ hospitalizations (HR 0.80); shorter hospital stay (5.8 vs. 7.0 days)	Social work referrals: 10.3% vs. 0%	↓ ED visits (−22%); ↓ hospitalizations (−20%)
Smith et al. (26)	USA	Cluster randomized controlled trial	26 ambulatory oncology clinics in a large academic health center the Northwestern Medicine (NM)	Oncology	Epic Systems	PROMs integrated into EHR to support referrals and symptom identification	Patient adoption of cPRO ↑25 to 40%	↑ referral uptake (RR = 1.66, 95% CI: 0.79 to 3.48).	NR
Solomon et al. (17)	USA	Controlled interrupted time series	Academic medical center, USA	Rheumatology (RA)	NR	PROMs used to guide visit scheduling and clinical management	Flares captured by PROMs: 25.0% vs. 28.5%, *p* = 0.44; PRO scores stable	108 delayed-visit recommendations; 31 early-visit recommendations; 17.6% of delayed-visit recommendations implemented	Visit volume ↓: −2.7 visits (95% CI −9.3 to 4.0).
Zylla et al. (30)	USA	Prospective single-arm feasibility study	Community cancer center, Frauenshuh Cancer Center, HealthPartners/Park Nicollet, Minneapolis, Minnesota.	Oncology (advanced cancer)	Epic Systems	PROMs used for symptom reporting and monitoring	66% completion rate; 36% of surveys identified ≥1 severe symptom; limited additional clinical action	26% of flagged symptoms through PROMs resulted in triage action	NR

Most instruments were patient-completed PROMs. Two exceptions are noted: ASDAS is a composite index (patient-reported items plus an acute-phase reactant), and the Passive Digital Marker used with QDRS where QDRS itself was patient-completed via the portal in that trial, although the scale can also be informant-rated.

Included studies were published between 2016 and 2026, with eight of ten appearing from 2019 onward, corresponding to wider use of patient portals, real-time alerts, and EHR-embedded decision support.

We summarized all the outcomes evaluated in these studies in Table 1.

### 3.2 Summary of results by study outcome

Across the included studies, patient-level outcomes demonstrated improvements in symptom identification and monitoring, with some studies reporting reductions in anxiety, depression, emergency department visits, hospitalizations, and symptom burden (27, 29–31, 33). Clinician-level outcomes showed that EHR-integrated PROMs supported clinical decision-making through alerts, diagnostic evaluations, referrals, triage, and symptom monitoring, while also improving perceived efficiency and usability among clinicians (17, 26, 28–31, 33). At the system level, several studies reported improvements in clinic efficiency, including reduced wait times, shorter clinic visits, increased clinic capacity, fewer hospital admissions and emergency department visits, and enhanced workflow integration (11, 17, 27, 29, 31, 33). However, evidence for improvements in patient satisfaction, visit volume, and broader long-term clinical outcomes was mixed across studies (17, 26, 30, 32).

#### 3.2.1 Reduction in care utilization (clinic visits and hospitalizations)

The results from three studies showed that EHR-integrated PROMs were associated with reductions in acute healthcare use in oncology and surgical/spine populations. Natori et al. reported that the completion of PROMs was associated with a 22% reduction in emergency room visits and a 20% reduction in hospitalizations. Similarly, Cheville et al. noted that among 24,874 patients, 85% reported at least one clinically actionable symptom, and EHR-facilitated collaborative care led to statistically significant reductions in symptom burden (e.g., anxiety −0.12, depression −0.08) (Table 1), which were associated with improved downstream clinical outcomes and reduced need for escalation of care (27). Azad et al. found that clinic visit duration decreased from 127.9 ± 51.5 min to 117.0 ± 45.7 min after PROM integration and electronic capture rates increased to ∼98% (11).

#### 3.2.2 Improved symptom control and clinical outcomes

PROMs integration enabled quantifiable improvements in symptom monitoring and management. Cheville et al. reported reductions across symptom domains: Anxiety: −0.12 (95% CI: −0.19 to −0.05), Depression: −0.08 (−0.15 to −0.01) (27). In Zylla et al., 36% of completed surveys reported at least one severe symptom, enabling identification of high symptom burden in real time, although many were already under management (30). Garcia et al. reported that over 50% of assessments triggered at least one clinical alert, with 41.8% specifically identifying nutritional needs, highlighting the breadth of symptom and supportive care detection (31).

#### 3.2.3 Changes in healthcare utilization and follow-up patterns

PROMs influenced visit frequency, scheduling, and care delivery models. Chan et al. reported that PROMs completion increased from 25% (paper) to 66% (electronic). In addition, they reported 1,500 clinic hours saved annually, and improvement in new-to-follow-up ratio from 1:3.1 to 1:2.2 resulting in shorter wait times and increased clinic capacity (33). Solomon et al. reported that monthly visit volume changed from 31.2 to 36.8 visits (intervention) vs. 30.4 to 38.7 (control) however no significant change in visit utilization despite PROM-guided scheduling decisions (17).

#### 3.2.4 Increased diagnostic activity and early detection

PROM integration was associated with measurable increases in diagnostic decision-making in one article. Boustani et al. (28) reported that the incidence of dementia diagnoses increased from 12.4% in usual care to 15.4% in the PROMs and EHR intervention group (AOR = 1.31, 95% CI: 1.05 to 1.64). Additionally, rates of diagnostic evaluations, including laboratory testing and neuropsychological assessments, increased from 29.0% to 36.7%**,** indicating greater clinical follow-up based on PROM data.

#### 3.2.5 Limited impact on clinic-level outcomes

Three studies reported minimal or no measurable changes in clinical outcomes or care experience following PROMs integration. Harle et al. (32) found no significant differences in patient or provider satisfaction scores between intervention and control groups, indicating no change in perceived care quality. Similarly, Solomon et al. (17) reported no significant differences in visit volume, flare rates, or delays in care despite the use of PROM-guided visit scheduling. In Smith et al. (26), clinician referral adoption increased, although this was not statistically significant. In Zylla et al. (30), PROMs implementation demonstrated good uptake, with 66% survey completion and 36% of surveys identifying severe symptoms; however, these findings resulted in limited additional clinical action, as many symptoms were already being managed.

### 3.3 Summary of EHR platforms and PROMs used

A wide range of PROMs were used across the included studies, primarily administered as self-reported measures through electronic platforms (e.g., EPIC, MyChart, Oracle Health Millennium). Table 1 shows the type of PROMs and end EHR platform used in each study. A comprehensive summary of the PROMs used (including type, number of items and domains, method of completion and description of domains) is presented in Supplementary Appendix C.

PROMs differed widely in type, length, and domains assessed, with both generic and condition-specific instruments used across studies. Patient-Reported Outcomes Measurement Information System (PROMIS)—based measures, including both fixed short forms and computer adaptive tests (CATs), were the most commonly used tools and were implemented across multiple platforms to assess a broad range of health domains (27, 31). Other commonly used generic PROMs include the Functional Assessment of Chronic Illness Therapy–Fatigue (FACIT-F), and Work Productivity and Activity Impairment (WPAI). In contrast, condition-specific PROMs were more commonly used in rheumatology and musculoskeletal settings, including measures such as Hospital Anxiety and Depression Scale (HADS), RADAI, RAPID-3, BASDAI, ASDAS, PSAID-12, ODI, and NDI, as well as oncology-specific symptom monitoring tools such as PRSM and SPADE.

Across studies, the most frequently assessed domains included pain, fatigue, physical function, anxiety, and depression, with several PROMs also evaluating broader constructs such as social participation, work productivity, and disease-specific symptom burden. Mental health and pain-related outcomes were consistently represented across both generic and condition-specific measures.

PROMs selection and implementation approaches varied considerably, with instruments ranging from brief single-item scales, such as numeric rating scales (NRS) and visual analogue scales (VAS), to multidimensional tools assessing numerous domains.

## 4 Discussion

### 4.1 Principal findings

This systematic review synthesized evidence from ten studies examining the integration of electronic patient-reported outcome measures (ePROMs) into electronic health record (EHR) workflows for chronic disease management in routine care. Overall, across the included studies, EHR-integrated PROMs appeared to improve several process-related outcomes, particularly symptom identification and aspects of clinic efficiency. However, the impact on longer-term clinical outcomes, patient satisfaction, and healthcare utilization remains inconsistent across studies. These differences indicate that while process improvements are relatively consistent, the impact on downstream clinical outcomes remains uncertain.

Three main patterns were observed across the included studies. First, several studies reported reductions in healthcare utilization or improvements in clinic efficiency, such as fewer follow-up visits, shorter wait times, and more efficient use of clinical resources. Second, integrating PROMs into clinical workflows improved the identification and monitoring of symptoms, allowing clinicians to recognize patient concerns earlier and respond more proactively. Third, several studies reported limited or non-significant improvements in patient satisfaction, visit volume, and overall clinical outcomes. Across the included studies, EHR-integrated PROMs were most consistently associated with improvements in process-related outcomes, particularly symptom identification and aspects of clinic efficiency; however, evidence for consistent improvements in patient outcomes, healthcare utilization, and other longer-term clinical outcomes remains mixed across studies.

### 4.2 Interpretation in context of data synthesis

The observed variability in outcomes is likely influenced by the substantial heterogeneity across studies. This heterogeneity likely contributed to differences in reported outcomes, limiting comparability across studies, and precluding meta-analysis. Moreover, the way in which PROMs were implemented also appeared to influence their effectiveness. Studies that used more active integration strategies, such as real-time alerts, automated triage systems, or clinical decision support tools, were more likely to report positive outcomes than those that collected PROM data passively without clear integration into care processes (31, 33). Together, these findings suggest that PROM integration may be most effective when patient-reported data actively informs clinical care through mechanisms such as real-time alerts, automated triage systems, and decision support tools, rather than functioning solely as a passive data collection process.

Outcomes also appeared to differ across clinical settings. Oncology-based studies more consistently demonstrated improvements in symptom detection and management, possibly because symptom monitoring is already well integrated into cancer care and patients undergoing active treatment or in the survivorship stage often experience more severe or rapidly changing symptoms (34, 35). In contrast, studies conducted in primary care and rheumatology more frequently reported improvements in clinic efficiency and resource utilization, suggesting that the benefits of EHR-integrated PROMs may differ depending on the clinical setting and patient population (16, 17, 36). Together, these findings suggest that the effectiveness of PROM integration may be highly context-dependent rather than universally reproducible across care settings.

Technological generation is a further source of heterogeneity. EHR systems in the mid-2010s typically supported electronic capture and display, whereas later implementations more often included patient-portal administration, computerized adaptive testing, automated alerts, and triage or decision-support rules. Improvements in data completeness, interoperability, and accessibility over this period may have influenced both the reliability of PROM records and the outcomes observed. Because most included studies date from 2019 onward, findings should be interpreted primarily in the context of contemporary EHR platforms rather than earlier systems.

Because quality was uneven across designs, more weight is given to process findings that recurred across studies than to single-study estimates of clinical or utilization effects.

### 4.3 Comparison with existing literature

Our findings align with prior literature demonstrating that electronic PROM collection is feasible and can enhance symptom detection and patient–clinician communication (12, 37). Reviews focused on oncology populations have similarly reported improvements in symptom monitoring and, in some cases, survival or healthcare utilization outcomes when PROMs are actively integrated into care. This systematic review extends the existing literature by specifically examining PROMs integrated within EHR workflows in routine care for patients with chronic conditions. Much of the previous research has focused on standalone electronic PROM collection systems or the general feasibility of PROM use, whereas this review focused on how PROMs are incorporated into clinical workflows and used by clinicians to support real-time care and decision-making. Our findings suggest that although improvements in symptom detection were generally consistent with previous literature, the effects on healthcare utilization and broader clinical outcomes were more modest and variable than those reported in some non-EHR-based PROM interventions. These differences may be related to how PROMs were implemented across studies. Interventions that actively incorporated PROM data into clinical care through alerts, triage systems, or clinician review appeared more likely to report benefits than studies where PROMs were collected without a clear process for follow-up or clinical action (38).

### 4.4 Strengths and limitations

This review followed a rigorous systematic methodology, including comprehensive database searching and adherence to PRISMA guidelines. The focus on EHR-integrated PROMs in routine care enhances the relevance of findings for real-world clinical practice. Additionally, the inclusion of patient partner perspectives strengthens the applicability and patient-centeredness of the review.

However, several limitations should be considered. A clarification of eligible designs was made after full-text screening and is a refinement of the registered protocol: the intervention (EHR-integrated PROMs in routine chronic care) and required outcomes were unchanged; the list of acceptable study designs was expanded to reflect how this intervention has been evaluated.

The relatively small number of included studies (*n* = 10), combined with a predominance of studies conducted in U.S.-based academic centers, may reduce how generalizable the findings are to other healthcare systems. Substantial heterogeneity in study design, technological generation, PROMs selection, and outcome measures precluded meta-analysis and limits direct comparability across studies. Many studies used observational or quality improvement designs, introducing potential risk of bias. Additionally, follow-up periods were often short, limiting the ability to detect longer-term impacts. Certain chronic conditions were also underrepresented, further constraining the breadth of conclusions. A small number of included intervention packages used composite or mixed measures or paired a PROM with a non-PROM digital tool.

Additionally, certain patient populations were overrepresented within the included studies. For example, five of the studies focused on oncology populations, which may have influenced the findings. As oncology-based studies appeared to demonstrate more consistent improvements in symptom detection, a more heterogeneous sample of studies may have resulted in even fewer observed downstream effects on broader patient outcomes.

### 4.5 Implications for clinical practice and health systems

Our findings suggest that EHR-integrated PROMs in relation to clinical decision making and action can offer meaningful benefits in routine care, particularly in enhancing symptom surveillance and improving aspects of clinic efficiency with relatively low additional burden (31, 33). The data analyzed in this systematic review indicate that their greatest value appears to be in supporting triage, remote monitoring, and early identification of patient needs, particularly in populations with high symptom burden or complex care requirements like oncology populations (27, 28).

Successful implementation requires careful attention to workflow integration. Features such as automated alerts, clinician-facing dashboards, and decision support tools appear important to maximizing impact, while poorly integrated systems risk contributing to alert fatigue or underutilization (28, 33). At the system level, PROMs to EHR integration may offer opportunities to improve capacity and reduce wait times, particularly in high-demand settings, though further evidence is needed to confirm these effects.

### 4.6 Implications for future research

Future research should prioritize larger, multi-center randomized controlled trials with longer follow-up periods and standardized outcome measures to better assess the impact of EHR-integrated PROMs on clinical and system-level outcomes. There is a need for studies in a broader range of chronic conditions and healthcare settings, particularly outside oncology and U.S.-based systems. Additionally, future research should examine the role of socioeconomic factors in access to and engagement with electronic PROMs, as not all patients have equal access to the digital technologies required to complete these assessments. Greater inclusion of diverse populations and healthcare systems outside of the United States would provide a more comprehensive understanding of the effectiveness and sustainability of EHR-integrated PROMs across different care settings. Research should prioritize patient-important outcomes, including equity considerations such as the digital divide, to ensure that PROMs integration does not exacerbate existing disparities. Finally, greater clarity is needed regarding optimal PROMs selection and integration strategies, including the use of computerized adaptive testing, alert thresholds, and clinician-facing dashboards.

## 5 Conclusion

This systematic review highlights the still limited but growing evidence surrounding the use of EHR-integrated PROMs for clinical decision-making and real-time monitoring in patients with chronic conditions. The findings suggest that this type of integration may be beneficial in certain clinical settings, including oncology, rheumatology, and primary care, where improvements were observed in areas such as symptom monitoring, wait times, and clinician–patient decision-making. Several studies also demonstrated that integrating PROMs into routine workflows can support more timely identification of patient concerns and allow for more immediate clinical follow-up and intervention. However, the evidence remains variable across outcomes and settings. Because the included studies differed in clinical population, purpose of PROM use, and depth of EHR integration, the findings should be read as context-dependent. Moreover, these findings also reflect a relatively recent generation of EHR platforms (primarily 2016–2026, and mostly 2019 onward). They should not be assumed to apply equally to earlier electronic record systems that lacked patient portals, real-time alerts, or clinical decision support. Process benefits (symptom identification, clinic efficiency) were most consistent in oncology and in implementations that used real-time alerts or triage; effects on satisfaction, visit volume, and longer-term clinical outcomes were mixed and cannot be generalized across settings. A single overall conclusion is therefore not warranted. Further research involving more diverse patient populations and health care systems is needed to better understand the overall effectiveness and real-world applicability of EHR-integrated PROMs in chronic disease management.

## Data Availability

The original contributions presented in the study are included in the article/Supplementary Material, further inquiries can be directed to the corresponding author.
