# Supplementary material for: Effectiveness of integrating patient-reported outcome measures into electronic health records for measurement-based care in patients with chronic illnesses: a systematic review
Source: Front Health Serv. 2026 Oct 14;6:1930126. doi: 10.3389/frhs.2026.1930126 (PMC13713690; doi:10.3389/frhs.2026.1930126)
Supplement: Supplementary file 1 [file Table1.docx]

**Appendix A. Information sources and full search strategies**

The search strategies used for each database are presented separately below, with the database-specific search syntax and results retained as reported in the original search records.

**Table A1. Databases and search dates**

| **Database** | **Interface / platform** | **Date searched** |
| --- | --- | --- |
| Ovid MEDLINE(R) ALL | Ovid | February 11, 2026 |
| Embase | Embase | February 11, 2026 |
| APA PsycInfo | Ovid | February 11, 2026 |
| Cochrane Library | Wiley | February 13, 2026 |
| CINAHL | EBSCO | February 11, 2026 |
| Web of Science | Science Citation Index | February 11, 2026 |
| Scopus | Elsevier | February 11, 2026 |

**Table A1.1** Ovid MEDLINE(R) ALL - January 1, 2000 to February 11, 2026

**Search Strategy**

| **Line** | **Search string** |
| --- | --- |
| 1 | patient reported outcome measures/ |
| 2 | "Quality of Life"/ and exp "Surveys and Questionnaires"/ |
| 3 | (HR-PRO or HRQL or HRQol or measurement based care or PROM or (patient report* adj3 (data or information or outcome*)) or (public report* adj3 (data or information or outcome*))).tw,kf. |
| 4 | ((QL or QoL or "quality of life") adj3 (index or indices or instrument* or measur* or scale* or tool*)).tw,kf. |
| 5 | 1 or 2 or 3 or 4 |
| 6 | exp Medical Records Systems, Computerized/ |
| 7 | exp Health Records, Personal/ |
| 8 | ((EHR* or EMR* or EPR* or PHR* or (automated or computer* or electronic)) adj3 (document* or record* or system*)).tw,kf. |
| 9 | ((personal health* or personal medical) adj3 (information* or record*)).tw,kf. |
| 10 | patient record* system*.tw,kf. |
| 11 | 6 or 7 or 8 or 9 or 10 |
| 12 | 5 and 11 |
| 13 | limit 12 to yr="2000 -Current" |
| 14 | animals/ not humans/ |
| 15 | 13 not 14 |
| 16 | limit 15 to (case reports or comment or editorial or letter) |
| 17 | 15 not 16 |
| 18 | limit 17 to "review articles" |
| 19 | 17 not 18 |
| 20 | limit 17 to (meta analysis or "scoping review" or "systematic review") |
| 21 | ((scoping or synthesis or systematic or umbrella) adj (overview or review)).tw,kf. |
| 22 | 17 and 21 |
| 23 | 19 or 20 or 22 |

**Table A1.2** Embase - January 1, 2000 to February 11, 2026

**Search Strategy**

| **Line** | **Search string** |
| --- | --- |
| 1 | *patient-reported outcome/ |
| 2 | exp *"quality of life"/ |
| 3 | *health care survey/ |
| 4 | (HR-PRO or HRQL or HRQol or measurement based care or PROM or (patient report* adj3 (data or information or outcome*)) or (public report* adj3 (data or information or outcome*))).tw,kf. |
| 5 | ((QL or QoL or "quality of life") adj5 (index or indices or instrument* or measur* or scale* or tool*)).tw,kf. |
| 6 | 1 or 2 or 3 or 4 or 5 |
| 7 | electronic medical record system/ |
| 8 | electronic medical record/ |
| 9 | electronic patient record/ |
| 10 | electronic health record/ |
| 11 | ((EHR* or EMR* or EPR* or PHR* or (automated or computer* or electronic)) adj3 (document* or record* or system*)).tw,kf. |
| 12 | ((personal health* or personal medical) adj3 (information* or record*)).tw,kf. |
| 13 | patient record* system*.tw,kf. |
| 14 | 7 or 8 or 9 or 10 or 11 or 12 or 13 |
| 15 | 6 and 14 |
| 16 | limit 15 to yr="2000 -Current" |
| 17 | animals/ not human/ |
| 18 | 16 not 17 |
| 19 | limit 18 to (books or chapter or "clinical trial (clinicaltrials.gov)" or conference abstract or "conference review" or editorial or letter or "preprint (unpublished, non-peer reviewed)") |
| 20 | 18 not 19 |
| 21 | limit 20 to (book or book series or clinical trials repository or multi-volume reference works or "preprint archive (unpublished, non-peer reviewed)" or trade journal) |
| 22 | 20 not 21 |
| 23 | limit 22 to "review" |
| 24 | 22 not 23 |
| 25 | limit 22 to (meta analysis or "systematic review") |
| 26 | ((scoping or synthesis or systematic or umbrella) adj (overview or review)).tw,kf. |
| 27 | 22 and 26 |
| 28 | 24 or 25 or 27 |

**Table A1.3** APA PsycInfo - January 1, 2000 to February 11, 2026

**Search Strategy**

| **Line** | **Search string** |
| --- | --- |
| 1 | patient reported outcome measures/ or self-report/ |
| 2 | "quality of life"/ or "health related quality of life"/ |
| 3 | "health and well being measures"/ or "quality of life measures"/ |
| 4 | (HR-PRO or HRQL or HRQol or measurement based care or PROM or (patient report* adj3 (data or information or outcome*)) or (public report* adj3 (data or information or outcome*))).tw,id. |
| 5 | ((QL or QoL or "quality of life") adj5 (index or indices or instrument* or measur* or scale* or tool*)).tw,id. |
| 6 | 1 or 2 or 3 or 4 or 5 |
| 7 | electronic health records/ |
| 8 | ((EHR* or EMR* or EPR* or PHR* or (automated or computer* or electronic)) adj3 (document* or record* or system*)).tw,id. |
| 9 | ((personal health* or personal medical) adj3 (information* or record*)).tw,id. |
| 10 | patient record* system*.tw,id. |
| 11 | 7 or 8 or 9 or 10 |
| 12 | 6 and 11 |
| 13 | limit 12 to yr="2000 -Current" |
| 14 | limit 13 to ("0200 book" or "0240 authored book" or "0280 edited book" or "0300 encyclopedia" or "0400 dissertation abstract") |
| 15 | 13 not 14 |
| 16 | limit 15 to (chapter or "column/opinion" or "comment/reply" or dissertation or editorial or letter or review-book or review-media or review-software & other) |
| 17 | 15 not 16 |

**Table A1.4** Cochrane Library (Wiley) - January 1, 2000 to February 13, 2026

**Search Strategy**

| **Line** | **Search string** |
| --- | --- |
| #1 | MeSH descriptor: [Patient Reported Outcome Measures] explode all trees |
| #2 | MeSH descriptor: [Quality of Life] this term only |
| #3 | MeSH descriptor: [Surveys and Questionnaires] explode all trees |
| #4 | #2 AND #3 |
| #5 | (("HR-PRO" or HRQL or HRQol or "measurement based care" or PROM or (("patient reports" or "patient reported") NEAR/3 (data or information or outcome*)) or (("public reports" or "public reported") NEAR/3 (data or information or outcome*)))):ti,ab,kw (Word variations have been searched) |
| #6 | (((QL or QoL or "quality of life") NEAR/3 (index or indices or instrument* or measur* or scale* or tool*))):ti,ab,kw (Word variations have been searched) |
| #7 | #1 OR #4 OR #5 OR #6 |
| #8 | MeSH descriptor: [Medical Records Systems, Computerized] explode all trees |
| #9 | MeSH descriptor: [Health Records, Personal] explode all trees |
| #10 | (((EHR* or EMR* or EPR* or PHR* or (automated or computer* or electronic)) NEAR/3 (document* or record* or system*))):ti,ab,kw (Word variations have been searched) |
| #11 | ((("personal health" or "personal healthcare" or "personal medical") NEAR/3 (information* or record*))):ti,ab,kw (Word variations have been searched) |
| #12 | ((patient NEAR record* NEAR system*)):ti,ab,kw (Word variations have been searched) |
| #13 | #8 OR #9 OR #10 OR #11 OR #12 |
| #14 | #7 AND #13 |

Limit 2000-2026

**Table A1.5** CINAHL (EBSCO) - January 1, 2000 to February 11, 2026

**Search Strategy**

| **Line** | **Search string** |
| --- | --- |
| 1 | MH "Patient-Reported Outcomes" |
| 2 | MH "Quality of Life" AND (MH "Questionnaires+" OR MH "Surveys") |
| 3 | XB ("HR-PRO" or HRQL or HRQol or "measurement based care" or PROM or ("patient report*" NEAR/3 (data or information* or outcome*)) or ("public report*" NEAR/3 (data or information* or outcome*))) |
| 4 | XB ((QL or QoL or "quality of life") NEAR/3 (index or indices or instrument* or measur* or scale* or tool*)) |
| 5 | 1 or 2 or 3 or 4 |
| 6 | MH "Patient Record Systems+" |
| 7 | MH "Medical Records, Personal" |
| 8 | XB ((EHR* or EMR* or EPR* or PHR* or (automated or computer* or electronic)) NEAR/3 (document* or record* or system*)) |
| 9 | XB (("personal health*" or "personal medical") NEAR/3 (information* or record*)) |
| 10 | XB "patient record* system*" |
| 11 | 6 or 7 or 8 or 9 or 10 |
| 12 | 5 and 11 |

Limit: 2000-2026

**Table A1.6** Web of Science (Science Citation Index) - January 1, 2000, to February 11, 2026

**Search Strategy**

| **Line** | **Search string** |
| --- | --- |
| 1 | ("HR-PRO" or HRQL or HRQol or "measurement based care" or PROM or ("patient report*" NEAR/3 (data or information* or outcome*)) or ("public report*" NEAR/3 (data or information* or outcome*))) (Topic) or ((QL or QoL or "quality of life") NEAR/3 (index or indices or instrument* or measur* or scale* or tool*)) (Topic) |
| 2 | ((EHR* or EMR* or EPR* or PHR* or (automated or computer* or electronic)) NEAR/3 (document* or record* or system*)) (Topic) or (("personal health*" or "personal medical") NEAR/3 (information* or record*)) (Topic) or "patient record* system*" (Topic) |
| 3 | 1 and 2 |
| 4 | Limit: 2000-2026 and Document Type: Article or Review Article |

**Table A1.7** Scopus (Elsevier) - January 1, 2000 to February 11, 2026

**Search Strategy**

| **Line** | **Search string** |
| --- | --- |
| 1 | ("HR-PRO" or HRQL or HRQol or "measurement based care" or PROM or ("patient report*" W/3 (data or information* or outcome*)) or ("public report*" W/3 (data or information* or outcome*)) or (QL or QoL or "quality of life") W/3 (index or indices or instrument* or measur* or scale* or tool*)) [Title/Abstract/KW] |
| 2 | (EHR* or EMR* or EPR* or PHR* or (automated or computer* or electronic) W/3 (document* or record* or system*) or ("personal health*" or "personal medical") W/3 (information* or record*) or "patient record* system*") [Title/Abstract/KW] |
| 3 | 1 and 2 |
| 4 | Limit: 2000-2026 and Document Type: Article or Review Article |
