# Supplementary material for: Effectiveness of integrating patient-reported outcome measures into electronic health records for measurement-based care in patients with chronic illnesses: a systematic review
Source: Front Health Serv. 2026 Oct 14;6:1930126. doi: 10.3389/frhs.2026.1930126 (PMC13713690; doi:10.3389/frhs.2026.1930126)
Supplement: Supplementary file 2 [file Table2.docx]

**Appendix B. Quality Appraisal**

**Table B1. Qualitative Randomized Control Trials**

| Author | Is randomization appropriately performed? | Are the groups comparable at baseline? | Are there complete outcome data? | Are outcome assessors blinded to the intervention provided? | Did the participants adhere to the assigned intervention? | comments? |
| --- | --- | --- | --- | --- | --- | --- |
| Smith et al | Yes | Yes | Yes | No | Can't tell | Unblinded pragmatic trial; implementation strategy uptake was variable across clusters and timing was imprecise around crossover points. |
| Cheville et al | Yes | Yes | Yes | No | Yes | 77% responded to one PROM, 49.5% completed both PROMs |
| Boustani et al | Yes | Can't tell. | Yes | No | Yes | Notable demographic imbalance in PDM arm (26% Hispanic vs ~12% in others). QDRS completion rate was only 20.7%. |
| Harle et al | Yes | Yes | Can't tell | No | Can't tell | No details on the method of how randomization was performed. Patients and healthcare providers were aware of intervention. Out of 712 patients there is data for only 680 patients on satisfaction outcomes. Small trial (4 clinics, n=21 PCPs). |

**Table B2. Qualitative Non-Randomized Control Trials**

| Author | Are the participants representative of the target population? | Are measurements appropriate regarding both the outcome and intervention (or exposure)? | Are there complete outcome data? | Are the confounders accounted for in the design and analysis? | During the study period, is the intervention administered (or exposure occurred) as intended? | comments? |
| --- | --- | --- | --- | --- | --- | --- |
| Solomon et al | Can't tell | Yes | Yes | Yes | No | Out of 227 patients only 150 completed PRO. Single academic centre, predominantly White female cohort limits generalisability. Low clinician response to EHR alerts (17.6% delays acted on) |
| Natori et al | Yes | Yes | Can’t tell | Yes | No | 56.9% of patients are non-responders (did not complete PROMs). Retrospective design; 56.9% non-response limits assessment of fidelity. COVID-19 restrictions prevented in-clinic PRO completion. |
| Zylla et al | No | Yes | No | No | No | out of 320 patients 142 were included in the study, and no details on why the remaining were excluded. Only 56% of patients completed survey via MyChart. Small convenience sample (n=80), 96% White, single site |
| Azad et al | Yes | Yes | Can’t Tell | No | Yes | Patients' engagement in the design of project (PROMs identification) was described as "assessment of patient opinion" without any details. Patients without baseline QOL assessment on MyHealth, completed it by electronic tablet or was manually entered by clinic staff. |

**Table B3. Quantitative Descriptive Studies**

| Author | Is the sampling strategy relevant to address the research question? | Is the sample representative of the target population? | Are the measurements appropriate? | Is the risk of nonresponse bias low? | Is the statistical analysis appropriate to answer the research question? | comments? |
| --- | --- | --- | --- | --- | --- | --- |
| Chan et al | Yes | Yes | Yes | No | Yes | **Prospective longitudinal observational study. No control group. Approximately 52% of ePROM forms were not completed, which may introduce nonresponse bias.** |
| Garcia et al | Yes | No | Yes | No | Yes | 51.6% response rate with significant demographic disparities (under-representation of Black and Hispanic patients). improvement aims. |
