# Supplementary material for: Effectiveness of integrating patient-reported outcome measures into electronic health records for measurement-based care in patients with chronic illnesses: a systematic review
Source: Front Health Serv. 2026 Oct 14;6:1930126. doi: 10.3389/frhs.2026.1930126 (PMC13713690; doi:10.3389/frhs.2026.1930126)
Supplement: Supplementary file 3 [file Table3.docx]

| **Appendix C. Summary of PROMs** | | | | | |
| --- | --- | --- | --- | --- | --- |
| Name of PROM | Proxy vs Self Completed | Type of PROM | # Items | # Domains | Domain Descriptors |
| Patient-Reported Outcomes Measurement Information System (PROMIS) | Self-report | Generic | NR | 7 | Depression; Anxiety; Fatigue; Pain interference; Physical function; Social role participation |
| Sleep disturbance, Pain, Physical function, Anxiety, Depression, Energy (SPADE) | Self-report | Generic (cancer symptom monitoring) | 6 | 6 | Sleep disturbance; Pain; Anxiety; Depression; Energy/Fatigue; Physical function |
| Quick Dementia Rating System (QDRS) | Self-report | Condition-specific (Alzheimer’s Disease and Related Dementia) | 10 | 4 | Cognitive; Functional; Behavioral; Psychological |
| Patient-Reported Outcomes Measurement Information System (PROMIS) | Self-report | Generic | 4–8 | 3 | Pain interference; Fatigue; Physical function |
| Rheumatoid Arthritis Disease Activity Index (RADAI) | Self-report | Condition-specific (Rheumatoid Arthritis) | 5 | 1 | Rheumatoid Arthritis disease activity |
| Routine Assessment of Patient Index Data 3 (RAPID-3) | Self-report | Condition-specific (Rheumatoid Arthritis) | 3 | 3 | Physical function; Pain; Patient global |
| Health Assessment Questionnaire (HAQ) | Self-report | Generic | 20 | 8 | Dressing; Arising; Eating; Walking; Hygiene; Reach; Grip; Activities |
| Bath Ankylosing Spondylitis Disease Activity Index (BASDAI) | Self-report | Condition-specific (Axial Spondyloarthritis) | 6 | 5 | Fatigue; Spinal pain; Joint pain; Enthesitis; Morning stiffness |
| Ankylosing Spondylitis Disease Activity Score (ASDAS) | Mixed (self + clinician) | Condition-specific (Axial Spondyloarthritis) | 5 | 5 | Pain; Duration of morning stiffness; Patient Global Assessment; Peripheral Pain/Swelling; Acute Phase Reactant |
| Psoriatic Arthritis Impact of Disease (PSAID-12) | Self-report | Condition-specific (Psoriatic Arthritis) | 12 | 12 | Pain;  Fatigue;  Skin problems;  Work and/or leisure activities;  Functional capacity;  Discomfort;  Sleep disturbance;  Coping;  Anxiety, fear, and uncertainty;  Embarrassment and/or shame;  Social participation;  Depression |
| Numeric Rating Scale (Pain) | Self-report | Generic | 1 | 1 | Pain intensity |
| Name of PROM | Proxy vs Self Completed | Type of PROM | # Items | # Domains | Domain Descriptors |
| Hospital Anxiety and Depression Scale (HADS) | Self-report | Generic | 14 | 2 | Anxiety; Depression |
| Functional Assessment of Chronic Illness Therapy – Fatigue (FACIT-F) | Self-report | Generic | 13 | 1 | Fatigue impact |
| Work Productivity and Activity Impairment (WPAI) | Self-report | Generic | 6 | 4 | Absenteeism; Presenteeism; Productivity loss; Activity impairment |
| PROMIS CATs | Self-report | Generic | 4–12 (adaptive) | 5 | Anxiety; Depression; Pain; Fatigue; Physical function |
| Patient-Reported Symptom Monitoring (PRSM) | Self-report | Condition-specific (oncology) | NR | NR | NR |
| PROMIS CATs | Self-report | Generic | 4–12 (adaptive) | 5 | Fatigue; Pain; Function; Anxiety; Depression |
| PROMIS CATs | Self-report | Generic | 4–12 (adaptive) | 9 | Pain interference;  Pain behavior;  Fatigue;  Anxiety;  Depression;  Physical function;  Sleep disturbance;  Sleep-related impairment;  Anger |
| Patient Health Questionnaire-9 (PHQ-9) | Self-report | Condition- Specific (mental health) | 9 | 1 | Depression symptoms |
| Oswestry Disability Index (ODI) | Self-report | Condition-specific (low back pain) | 10 | 10 | Pain Intensity;  Personal Care;  Lifting;  Walking;  Sitting;  Standing;  Sleeping;  Sex Life;  Social Life;  Traveling |
| Neck Disability Index (NDI) | Self-report | Condition-specific (neck pain) | 10 | 10 | Pain Intensity; Personal Care; Lifting; Reading; Headaches; Concentration; Work; Driving; Sleeping; Recreation |
| Visual Analogue Scale (VAS) (Pain) | Self-report | Generic | 1 | 1 | Pain intensity |
